# Molecular Insights into Royal Jelly Anti-Inflammatory Properties and Related Diseases

**DOI:** 10.3390/life13071573

**Published:** 2023-07-17

**Authors:** Lilla Bagameri, Sara Botezan, Otilia Bobis, Victorita Bonta, Daniel Severus Dezmirean

**Affiliations:** Department of Apiculture and Sericulture, Faculty of Animal Sciences and Biotechnologies, University of Agricultural Sciences and Veterinary Medicine Cluj-Napoca, 400372 Cluj-Napoca, Romania; lilla.bagameri@usamvcluj.ro (L.B.); sara.botezan@student.usamvcluj.ro (S.B.); victorita.bonta@usamvcluj.ro (V.B.); ddezmirean@usamvcluj.ro (D.S.D.)

**Keywords:** royal jelly, anti-inflammatory activity, antioxidant activity, natural products, anticancer potential, biological activity

## Abstract

Royal jelly (RJ), a highly nutritious natural product, has gained recognition for its remarkable health-promoting properties, leading to its widespread use in the pharmaceutical, food, and cosmetic industries. Extensive investigations have revealed that RJ possesses a broad spectrum of therapeutic effects, including anti-inflammatory, antioxidant, antitumor, anti-aging, and antibacterial activities. Distinctive among bee products, RJ exhibits a significantly higher water and relatively lower sugar content. It is characterized by its substantial protein content, making it a valuable source of this essential macronutrient. Moreover, RJ contains a diverse array of bioactive substances, such as lipids, phenolic compounds, flavonoids, organic acids, minerals, vitamins, enzymes, and hormones. This review aims to provide an overview of current research on the bioactive components present in RJ and their associated health-promoting qualities. According to existing literature, these bioactive substances hold great potential as alternative approaches to enhancing human health. Notably, this review emphasizes the anti-inflammatory properties of RJ, particularly in relation to inflammatory diseases, such as multiple sclerosis (MS), rheumatoid arthritis (RA), and inflammatory bowel diseases (IBD). Furthermore, we delve into the antitumor and antioxidant activities of RJ, aiming to deepen our understanding of its biological functions. By shedding light on the multifaceted benefits of RJ, this review seeks to encourage its utilization and inspire further investigation in this field.

## 1. Introduction

Infections, chemical toxins, mechanical injuries, and many other factors can cause inflammation, a natural defense mechanism. When bacteria invade the organism, host cells are driven to secrete pro-inflammatory cytokines. Employing natural remedies as a treatment has received special attention recently because taking anti-inflammatory pharmaceuticals may have a variety of negative side effects [1].

Royal jelly (RJ) is a yellowish-white, creamy substance secreted by the hypopharyngeal and mandibular glands of worker bees. It serves as the primary nourishment for developing larvae during their initial three days and continues to be the exclusive food source for honeybee queens throughout their lives. Recognized for its exceptional nutritional value, RJ has gained the reputation of a “superfood” with numerous potential health benefits for human consumption. It is characterized by its creamy texture and has a pH range of 3.6–4.2. It comprises a diverse range of essential components, including proteins, lipids, carbohydrates, minerals, amino acids, vitamins, enzymes, and hormones. These constituents contribute to its remarkable nutritional profile and may play a role in its physiological effects [2,3].

With an estimated yearly output of more than 4000 tons of RJ, China is regarded as the world’s greatest producer and exporter, accounting for over 90% of the total amount collected globally [4,5]. Several countries have created their own national standards for the quality requirements of RJ [6].

Due to its health benefits, RJ has been used as an alternative treatment since ancient times and was especially widespread in Asia and Ancient Egypt [6]. In recent years, there has been a significant rise in interest in the food sector, with the significance of RJ and its distinct pharmacological and therapeutic qualities being highlighted. RJ meets functional requirements for dietary supplements by possessing anti-inflammatory, anticancer, antioxidant, hypotensive, anti-aging, and anti-microbial activities [5]. Considering these therapeutic properties of RJ, the present review aims to highlight recent findings on its activity against inflammatory diseases.

## 2. Chemical Composition

RJ’s rich composition not only holds potential for pharmaceutical applications but also for use in dietary supplements and functional foods, aiding in overall health and well-being [5]. As the critical sustenance for queen bees, RJ necessitates the inclusion of all life-supporting nutrients, encompassing sugars, proteins, lipids, and water, harmoniously proportioned. RJ stands as the bee product with the most significant water content, accounting for 60–70%, while exhibiting a lower sugar ratio, 7–16%, relative to other bee derivatives. It also manifests a high protein content, spanning 10–18%, and lipids ranging between 3–8%. The composition extends to phenolic compounds, flavonoids, organic acids, minerals, vitamins, enzymes, and hormones [3,5,7,8,9,10].

### 2.1. Proteins and Amino Acids

Proteins are a major component of RJ, representing more than 50% of its dry matter [6]. Major royal jelly proteins (MRJPs) and specific peptides, namely jeleines, royalsin, royalactin, apidaecin, and defensin-1, constitute the protein composition of RJ [11]. There have been nine significant royal jelly proteins (MRJP1-9) distinguished by several researchers [3,12,13], each with molecular weights spanning between 49 to 87 kDa. Among these, MRJP1 and MRJP3 contribute the most significant proportions, accounting for 31% and 26%, respectively. They are closely followed by MRJP2 and MRJP5 [14]. Expanding our knowledge of protein dynamics, a groundbreaking study by Buttstedt [15] brought to light the unique role of 10-Hydroxy-Δ2-decenoic acid (10-HDA) in manipulating the structure of a particular protein ensemble found in honeybee (*Apis mellifera*) RJ. This complex consists of three key elements: MRJP1, apisimin, and 24-methylenecholesterol. Buttstedt’s research illustrates how the presence of 10-HDA influences this complex, encouraging the formation of fibrils—a process with potential implications for the functionality of RJ. The revelations from this research open new avenues in the world of protein studies and provide valuable insights for fields such as entomology and apiculture [15]. In their 2019 research, Mureşan and Buttstedt [16] investigated the stability of MRJPs in the context of varying pH environments. They found a direct correlation between the pH levels and the stability of these proteins, influencing their digestion process. The study further revealed that the beneficial aspects of these proteins, such as their anti-inflammatory properties, are reliant on this pH-dependent stability [16].

In addition to these proteins and peptides, RJ also encompasses a collection of free amino acids, which includes lysine, proline, cystine, aspartic acid, valine, glutamic acid, serine, glycine, cysteine, threonine, alanine, tyrosine, phenylalanine, leucine, isoleucine, and glutamine [17,18].

### 2.2. Carbohydrates

RJ is recognized for its complex and diverse nutrient content, which encompasses an array of carbohydrates. These include glucose, fructose, sucrose, maltose, turanose, trehalose, and isomaltose [19]. The presence and distribution of these carbohydrates in RJ can be a crucial factor in determining its authenticity [11]. The carbohydrate content in RJ appears to be influenced by various factors such as the harvesting season, the species of bees, and the geographical origin of the product [20]. Consequently, these variables could potentially serve as distinctive markers in authenticity tests for RJ products, providing valuable insights for both food science researchers and the apiculture industry [11].

### 2.3. Lipids and Fatty Acids

RJ is a remarkable substance, rich in a range of lipids. Its lipid composition is predominantly composed of medium-chain fatty acids, among which 10-HDA is a major constituent [10,21]. This fatty acid alone accounts for a substantial portion of RJ’s lipidic profile. It is regarded as a freshness and quality marker [10]. However, other fatty acids, such as sebacic acid and 9-hydroxy-2-decenoic acid [5,10], are also present and contribute to the overall lipid matrix of RJ. Beyond fatty acids, RJ also comprises other lipid-soluble substances, including waxes, sterols, and phospholipids [22]. Due to the wide variety and complexity of lipids found in RJ, a recent study [23] utilized sophisticated techniques, such as gas chromatography (GC) and ultra-high performance liquid chromatography paired with ion mobility-quadrupole-time-of-flight-mass spectrometry (UHPLC-IM-Q-TOF-MS). These methods offer high precision, sensitivity, and accuracy, making them highly effective in analyzing intricate mixtures of lipids.

The investigation was successful in identifying and quantifying nine distinct classes of lipids in RJ. These included phosphatidylcholine (PC), diacylglycerol (DG), lyso-PC (LPC), sphingomyelin (SM), triglycerides (TG), phosphatidylethanolamine (PE), ceramide (Cer), cardiolipin (CL), and lyso-PE (LPE). This research marks a significant step forward in understanding the intricate lipid composition of RJ, which, in turn, sheds light on its various biological activities and potential health benefits. Future investigations in this area could provide even more insights into the functional properties of RJ, contributing to its potential use in various health and nutritional applications.

Studies about the anti-inflammatory effects of fatty acids will be discussed in the “RJ as an adjuvant in anti-inflammatory diseases” section.

### 2.4. Other Constituents

RJ is characterized by its phenolic compounds, comprising substances such as pinobanksin, hesperetin, naringenin, isosakuranetin, chrysin, acacetin, luteolin, apigenin, kaempferol, isorhamnetin, formononetin, along with various glycosides. These compounds contribute to the biological activities and potential health benefits associated with RJ [20,24].

The vitamin profile of RJ is also noteworthy. It includes essential vitamins, such as biotin, riboflavin, thiamin, pantothenic acid, inositol, niacin, pyridoxine, and vitamin E. These vitamins are key to multiple biological functions, further enhancing the nutritional value and health-promoting potential of RJ [25]. This bee product also incorporates a range of minerals, such as potassium (K), phosphorus (P), calcium (Ca), magnesium (Mg), sodium (Na), zinc (Zn), chromium (Cr), and cadmium (Cd). The presence and concentration of these minerals in RJ are affected by a wide range of factors. These include environmental conditions, the time of the harvest season, and the unique biological traits of the bees. Therefore, these factors should be taken into account when studying the composition and properties of RJ [11,26,27].

A variety of factors significantly influence the production and chemical composition of RJ, as emphasized in the study conducted by Al-Kahtani and Taha [28]. They highlighted the pivotal role of the post-grafting period, demonstrating that it profoundly affects the output and macro and trace elements of this bee product. A precise post-grafting duration is hence critical for optimizing both quantity and quality. Harvest timing also wields substantial influence, with suboptimal timing potentially impacting both yield and chemical constitution. Al-Kahtani and Taha [29] sought to determine the optimal timing for harvesting RJ based on its yield and nutritional composition, examining three timeframes: 24, 48, and 72 h post-grafting. The results indicated that RJ collected after 72 h of grafting produced the highest yield per queen cell, contrasting with the minimum yield seen at the 24 h mark. The nutritional constitution of this bee product also exhibited a temporal dynamic. The 72 h harvest exhibited the lowest moisture levels and highest amounts of crude protein, ash, fructose, and glucose. RJ harvested at 24 h, however, showed the highest concentration of lipids. Furthermore, a trend towards decreasing pH and increasing acidity was observed over time. The study’s conclusions suggest a 72 h grafting period to maximize RJ yield and optimize nutritional content. Yet, it is important to recognize that the changing nutritional characteristics over time mean that RJ properties may vary depending on when it is harvested post-grafting [29]. Additionally, the dietary intake of the bee colonies directly affects the volume and biochemical composition of the RJ produced. Seasonal variations introduce another layer of complexity, resulting in oscillations in its production and chemical attributes. These findings highlight the multifactorial aspect of RJ production and emphasize the need for a comprehensive understanding to optimize beekeeping practices [28].

## 3. RJ as an Adjuvant in Inflammatory Diseases

When the organism is challenged by pathogens, harmful toxins, irradiation, or microbial infections, inflammation occurs as a natural defense mechanism. Numerous physiological and immunological pathways are activated by this complex process; thus, when inflammation becomes dysregulated as a result of certain circumstances, it can harm nearby tissue and induce a wide range of diseases [30]. 

Characteristic inflammatory symptoms, including pain, redness, and heat, involve immune cells, such as B and T lymphocytes, monocytes, macrophages, neutrophils, and basophils, as well as mast and dendritic cells. The inflammatory response is largely influenced by the type of stimulus, all sharing similar mechanisms, such as the identification of pattern-recognition receptors on foreign pathogen’s cell surfaces or intracellular signaling caused by harmed cells or tissues [31].

**Figure 1:**
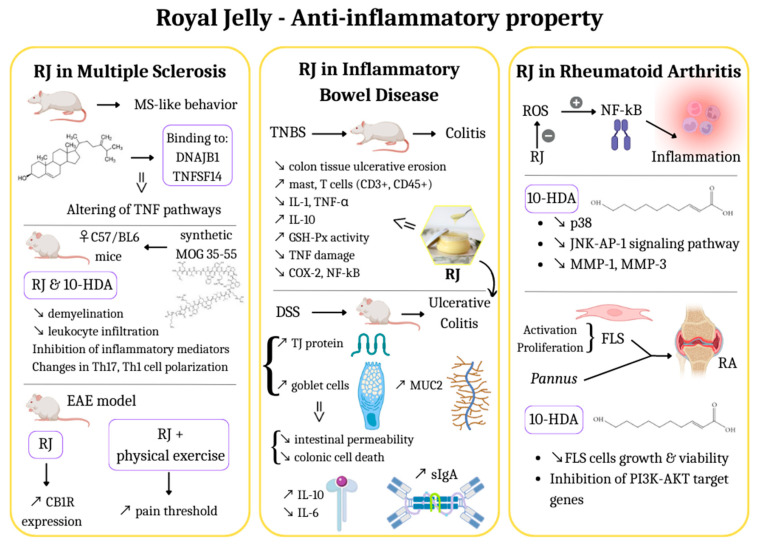
RJ—Mechanisms of action in inflammatory-related diseases. First panel: RJ in Multiple Sclerosis (MS); Second panel: RJ in Inflammatory Bowel Disease (IBD); Third panel: RJ in Rheumatoid Arthritis (RA). ↗ increase, ↘ decrease (Created with canva.com, accessed on 22 April 2023).

Nowadays, a wide range of anti-inflammatory medications on the market work by preventing the production of prostaglandins, but they have substantial adverse effects as well [31]. As a result, several studies investigating natural products with the potential to enhance anti-inflammatory effects without having negative side effects have been carried out, and various natural substances with the potential to enhance immune function have been found. It is worth noting that these natural products have a beneficial effect on both the human body and animals. As an example, a natural product called RJ has been traditionally used to enhance immune system performance [32]. Numerous biological and chemical factors, including cytokines, pro-inflammatory enzymes, as well as the enzymatic degradation of tissues, all contribute to the inflammatory process. Interleukin-1 (IL-1), interleukin-6 (IL-6), and tumor necrosis factor-alpha (TNF-α) production are effectively inhibited by RJ treatment in a dose-dependent manner without having any deleterious effects on macrophages in vitro. In autoimmune disorders, such as rheumatoid arthritis (RA) and inflammatory bowel diseases (IBD), RJ may play a crucial role in enhancing life quality [5]. Figure 1 represents a summary of the information described in the text regarding three important inflammatory-related diseases taken into discussion.

### 3.1. RJ in Multiple Sclerosis

Multiple sclerosis (MS) is an inflammatory condition that affects the nervous system and is thought to be immunologically mediated and persistent. This disease comes with a damaged blood–brain barrier, lymphocytes, microglia, and macrophages being attracted to the lesion sites. It is distinguished by the destruction of fatty myelin sheaths around the brain and spinal cord’s axons, which results in demyelination along with a wide range of symptoms. Although genetic and environmental variables have been demonstrated to play an essential role, the fundamental etiology of MS remains unclear [33].

A descriptive analysis performed by Podbielska et al. [34] indicated that lipids could potentially play a key role in the immunopathogenesis of MS while also being involved in the progression and remission of the disease.

Natural product-based therapies have shown promising outcomes in the treatment of MS symptoms and the disease’s progression. Due to its antioxidant, anti-inflammatory, and anti-apoptotic properties, RJ is used to treat a variety of diseases, including neurological disorders. RJ has been linked to relieving chronic pain, neuroplasticity, and altering neurotransmitters involved in anxiety and depression conditions [35,36].

Lohrasbi et al. [35] pointed out that rats with MS-like behaviors, supplemented with 100 mg/kg/day of RJ, could show an improvement in terms of mobility, pro-inflammatory cytokine functions, and demyelination. Researchers highlighted 10 bioactive RJ compounds with suitable binding affinities, including Vitamin A, Vitamin B2, Vitamin E, hesperetin, kaempferol, naringenin, formononetin, genistein, isosakuranetin, and 24-methylenecholesterol. Based on the artificial intelligence analysis (AI) study, they discovered that these bioactive substances might bind to DNAJB1 and TNFSF14 protein-coding genes. According to the molecular docking analysis of bioactive chemicals, RJ consumption may alter important molecular signaling pathways, improving life quality and increasing muscular strength in patients with MS. Based on their new findings, researchers revealed that 24-methylenecholesterol, a low-energy molecule, is bound to the surfaces of the TNFSF14 and DNAJB1. These results suggest that TNF pathways were altered because of consuming this natural bee product [35]. 

As Cannabinoid-1-receptors (CB1R) are therapeutic targets for the treatment of various associated symptoms of MS, Kheirdah et al. [36] aimed to assess the impact of aerobic exercise and two doses of RJ on hippocampus CB1R and pain threshold (PT) in an EAE model. RJ was administered intraperitoneal at dosages of 50 and 100 mg/kg/day throughout a five-week period of exercise training (ET), which was completed four times per week at different speeds ranging between 11 to 15 m/min for 30 min. In rats with EAE, endurance training had no remarkable impact on PT or hippocampus CB1R. In comparison to the EAE group, the CB1R gene expression levels in the RJ100 group were increased. Additionally, a higher PT level was observed in the ETRJ50 and ETRJ100 groups compared to the EAE group. PT and CB1R were more significantly affected by the combination of ET and RJ50 than by either of them alone. These findings indicate that RJ at 50 or 100 mg/kg doses should be consumed, and this supplementation should be combined with physical exercise for better results [36].

Another study investigated the effects of RJ on female C57/BL6 mice with induced experimental autoimmune encephalomyelitis (EAE), a model for MS. The mice received daily assessments for 25 days after receiving synthetic MOG35-55. Histological analysis using techniques such as H&E and LFB staining, BrdU incorporation, ELISA, and Real-time PCR were employed to evaluate demyelination, proliferation, lymphocyte infiltration, cytokine profiles, and gene expression levels. The results indicated that RJ and 10-HDA prevented the development of EAE. The treated groups exhibited reduced demyelination, decreased leukocyte infiltration in the central nervous system, and dose-dependent inhibition of inflammatory mediators. RJ and 10-HDA were found to modulate the immune response by primarily affecting the polarization of Th17 and Th1 cells. These findings highlight the potential therapeutic benefits of RJ and 10-HDA in MS and support their use in the treatment of inflammatory diseases [37].

A randomized clinical trial conducted by Oshvandi et al. [38] aimed to assess the impact of RJ supplementation on the quality of life in patients diagnosed with MS. The study involved 100 MS patients, divided into an experimental group and a control group. The experimental group received a daily capsule of 500 mg of RJ for 90 days, while the control group received a placebo. The researchers utilized the MS-specific quality of life questionnaire and the Barthel Index of Daily Living Activities to evaluate patients' quality of life and daily activities before and after the intervention. The results demonstrated that the experimental group experienced a significantly higher average quality of life score after the intervention compared to the control group, even after accounting for potential confounding factors. Additionally, the experimental group exhibited a significant improvement in daily activity status scores compared to the control group. These findings suggest that incorporating RJ supplements into the daily routine can have a positive impact on the quality of life for MS patients [38].

In summary, the studies mentioned above provide insights into the potential benefits of RJ in the context of MS. The in vitro and in vivo experiments demonstrated improvements in mobility, pro-inflammatory cytokine functions, demyelination, and immune response modulation. The molecular docking analysis indicated the interaction of bioactive compounds with specific genes and molecular pathways related to MS. Furthermore, clinical data highlighted the positive effects of RJ supplementation on the quality of life and daily activities of MS patients. These findings support the exploration of this bee product as a potential therapeutic option for managing MS symptoms and disease progression (Figure 1, First panel).

### 3.2. RJ in Inflammatory Bowel Disease

Both Crohn’s disease (CD) and ulcerative colitis (UC) are chronic inflammatory bowel diseases (IBD), which share many of the same symptoms and cause inflammation in the digestive tract. Its incidence is ascribed to a number of circumstances, some of which include geographic location, low-quality food, genetics, and decreased immunological response [39].

Weight loss, fever, rectal bleeding, diarrhea, and stomach discomfort are some of the symptoms of CD and UC, inflammation being the fundamental characteristic of them. These disorders can affect men and women equally. The terminal ileum, cecum, perianal region, and colon are often affected by CD, although it can also affect any part of the intestine. In contrast, UC affects the rectum and can spread continuously across the entire colon or only a portion of it. The inflammation in UC is only present in the mucosa and submucosa with cryptitis and crypt abscesses, in contrast to the thickened submucosa, transmural inflammation, fissuring ulceration, and granulomas that are histologically present in CD [40].

In vitro studies have provided valuable insights into the anti-inflammatory properties of RJ and its components in the context of colitis. For example, Yang et al. [41] demonstrated in vitro that 10-HDA, a component isolated from RJ, can prevent the secretion of pro-inflammatory cytokines TNF-α, IL-1β, and IL-8 in WiDr adenocarcinoma cells. Additionally, they found that this fatty acid effectively increased the production of IL-1Ra (IL-1 receptor antagonist), which acted as a constraint on IL-1β production.

Moving to in vivo animal studies, the effects of RJ have been investigated in 2, 4, 6-trinitrobenzene sulfonic acid (TNBS)-induced colitis model. It was discovered that RJ supplementation reduced colon tissue ulcerative erosion and increased the presence of mast cells, CD3+, and CD45+ T cells. Furthermore, pre-treatment with RJ decreased the production of pro-inflammatory cytokines, such as IL-1 and TNF-α, while enhancing the production of the anti-inflammatory cytokine IL-10 in the colon. RJ was also found to improve plasma glutathione peroxidase (GSH-Px) activity, reduce TNF-α damage, and suppress the production of major inflammatory mediators, including COX-2 and NF-kB [3,42].

In another animal study conducted by Guo et al. [43], the protective effects of RJ were investigated in a DSS-induced UC model in mice. RJ supplementation in these mice increased the levels of tight-junction proteins, goblet cells, and mucin secretion (MUC2), leading to alleviation of symptoms, reduced intestinal permeability, and colonic cell death. Moreover, RJ supplementation resulted in increased expression of the anti-inflammatory cytokines IL-10 and IgA while decreasing the expression of the pro-inflammatory cytokine IL-6. The relative abundance of gut microbiota was also affected by DSS, with specific changes observed. Treatment with RJ increased the relative abundance of certain beneficial gut microbiota. These findings demonstrated that RJ could mitigate DSS-induced colitis by strengthening the intestinal mucosal barrier and modulating gut microbiota composition.

In conclusion, in vitro and in vivo animal studies collectively suggest the anti-inflammatory effects of RJ in colitis (Figure 1, Second panel). In vitro studies demonstrated the ability of RJ components, such as 10-HDA, to suppress the secretion of pro-inflammatory cytokines and enhance the production of anti-inflammatory cytokines. Animal studies showed that RJ supplementation reduced tissue damage, modulated immune response, strengthened the intestinal mucosal barrier, and influenced gut microbiota composition. These findings highlight the potential of RJ as a therapeutic option for managing colitis, but further research and clinical trials are necessary to fully understand the underlying mechanisms and optimize its clinical application.

### 3.3. RJ in Rheumatoid Arthritis

Rheumatoid arthritis (RA) is a chronic, autoimmune condition that seriously threatens healthspan, evolving gradually, affecting primarily elderly people [20]. It is frequently characterized by pain, edema, stiffness, inflammation, and joint damage. Chronic synovial membrane inflammation causes bone and cartilage deterioration, resulting in the degeneration of the afflicted joints. Inflammation of the blood vessels, internal organs, and tendon sheaths is among the most common symptoms, along with discomfort, exhaustion, and movement restrictions. Early recognition and the beginning of treatment as soon as possible are crucial as the immune process can be considerably altered if this condition is identified and treated within three to six months of the onset of symptoms [44]. Results of RA therapy (steroids and anti-TNF drugs) in the elderly are unsatisfactory due to age-related loss in organ function, comorbidities, and body composition changes [45]. 

Clinical investigations have indicated that oxidative stress plays a significant role in the genesis of RA, resulting in high levels of oxidative stress biomarkers and low antioxidant status. NF-kB is activated by rROS, which might lead to inflammatory reactions in RA. Thus, supplementation with RJ as an antioxidant agent may contribute to symptom relief and life quality enhancement. Nowadays, pharmacologic treatments for RA symptoms include non-steroidal anti-inflammatory drugs (NSAIDs), corticosteroids, and disease-modifying anti-rheumatic drugs (DMARDs), although these treatments come with certain adverse effects. As a result, complementary therapies, particularly nutritional supplements, have gained more attention [46].

In vitro studies were conducted to investigate the effects of 10-HDA, a compound found in RJ, on key signaling pathways and enzymes associated with RA. The researchers observed that this fatty acid significantly reduced the activity of p38, c-Jun N-terminal kinases-activating protein-1 (JNK-AP-1) signaling pathway, and matrix metalloproteinases (MMP-1, MMP-3) [5,47]. These findings suggested that 10-HDA may have a protective effect against the adverse effects of RA treatment.

Researchers sought to explore the impact of 10-HDA on fibroblast-like synoviocytes (FLS) cells, which play a crucial role in the pathogenesis of RA. Increased activation and proliferation of FLS cells, along with the development of pannus that invades nearby bone and cartilage, are characteristic features of RA. Inhibition of FLS cell growth and control of pannus formation are important therapeutic goals in RA treatment. Previous research indicated that 10-HDA could potentially reduce FLS cell growth [44]. However, recent findings highlighted the inverse relationship between dosage and time with regard to the viability and histone deacetylase (HDAC) activity of FLS cells. This discovery opened up new possibilities for the development of histone deacetylase inhibitors (HDACI) as a potential treatment option for RA. Consequently, 10-HDA was implicated in inhibiting the target genes of the phosphoinositide-3-kinase-protein kinase B/AKT (PI3K-AKT) pathway, thereby suggesting its potential as an alternative treatment option for RA [32] (Figure 1, Third panel).

Finally, clinical data is needed to validate the findings from in vitro studies. Clinical trials involving human participants with RA would be required to evaluate the efficacy and safety of 10-HDA as a therapeutic agent. These trials would assess the impact of this fatty acid on disease progression, symptoms, and other relevant factors. If the clinical data support the earlier findings, 10-HDA could potentially be considered as a complementary treatment option for RA.

## 4. Additional Anti-Inflammatory Effects of RJ

A group of researchers developed a personalized treatment based on the protease enzyme technique to hydrolyze RJ, aiming to compare the anti-inflammatory and immune-boosting properties of enzyme-treated RJ (ERJ) on macrophages and mice. They discovered that ERJ could affect macrophage proliferation and offer protection against LPS-induced stress. The mice selected for this study that were given ERJ for 4 weeks and stimulated with LPS presented considerably lower levels of TNF-α, IL-1, IL-6, IL-10, and IL-12, as well as IFN-γ. Moreover, B-lymphocyte and T-lymphocyte proliferation, as well as the activity of naturally occurring natural killer cells, were all markedly and dose-dependently boosted by ERJ. These findings show that ERJ possesses substantial anti-inflammatory and immune-promoting properties, making it a promising dietary ingredient for the treatment of inflammatory diseases [48].

The three main fatty acids found in RJ, namely 10-HDA, 10-hydroxydecanoic acid (10-HDAA), and sebacic acid (SEA), were examined for their anti-inflammatory properties by Chen et al. [49]. Whereas all of them exhibited significant, dose-dependent inhibitory effects on the release of the key inflammatory mediators (nitric oxide and IL-10), findings showed that only SEA had the ability to reduce the production of TNF-α. These RJ fatty acids have also been shown to affect a number of important inflammatory genes, with 10-HDA exhibiting different modulatory actions in comparison to the other two fatty acids. The authors also discovered that all of them possessed a regulatory effect on a number of proteins involved in the mitogen-activated protein kinase (MAPK) and nuclear factor kappa-light-chain-enhancer of activated B cells (NF-κB) signaling pathways [50]. Chen et al. [50] examined the in vitro anti-inflammatory effects of 10-HDA and noticed a reduction in the expression of crucial inflammatory genes, such as IL-1, IL-6, COX-2, and MCP-1. Mice were enrolled to examine the effects of 10-HDA on lung damage brought on by LTA. In accordance with the findings, 100 mg/kg of 10-HDA can have protective effects by limiting the production of inflammatory cytokines, including IL-10, MCP-1, and TNF-α. Even though the inhibitory effects of this fatty acid were dose-dependent, it was speculated to possess anti-inflammatory effects. Further investigations to comprehend this fatty acid’s accurate action mechanisms were suggested by the authors. 

RJ’s anti-inflammatory effects on the BV-2 murine microglial cell line after exposure to LPS were investigated by You et al. [51]. RJ was discovered to have a protective effect by reducing the inflammatory response; the underlying processes may be connected to pro-inflammatory cytokines production inhibition. RJ has the potential to drastically reduce the expression of the pro-inflammatory protein cyclooxygenase-2 (COX-2). Moreover, RJ can decrease inflammatory mediator levels by inhibiting the nuclear factor kappa B (NF-kB) and c-Jun N-terminal kinase (JNK) pathways [51]. Studies involving phytohaemagglutinin-activated peripheral blood mononuclear cells (PBMCs) as an in vitro model have shown that RJ fatty acids, including 3, 10-dihydroxy-decanoic acid (3,10-DDA) and 10-HDA at the concentration of 500 µM have an inhibitory effect on PBMC proliferation. Moreover, it suppressed Th1 and Th2 immune responses, along with modulating TNF-α and IL-1β production. While TNF-α and IL-1β levels were unaffected by 3,10-DDA at a concentration of 500 µM, the production of these cytokines by stimulated PBMCs was inhibited by the same amount of 10-HDA. The researchers concluded that RJ fatty acids had a substantial, dose-dependent immunomodulatory impact in vitro [52].

It was shown that RJ can stimulate the proliferation of healthy lymphocytes and has a stimulatory effect on interferon-gamma (IFN-γ) production. RJ treatment shifted the Th1/Th2 cytokine ratio in favor of the Th1 in patients with autoimmune Basedow Graves’ illness. This indicates that RJ may be beneficial in the treatment of Graves’ disease as an immunomodulatory agent and an antithyroid medication [53].

Arzi et al. [54] also examined the anti-inflammatory effects of RJ in formalin-induced rat paw edema. The researchers showed that RJ inhibited inflammation in a dose-dependent manner, considerably reducing edema at doses of 50 and 100 mg/kg. It was also demonstrated that there was no discernible difference in the inhibition of formalin-induced edema between groups treated with dosages of 50 and 100 mg/kg and the positive control group treated with aspirin 300 mg/kg. 

Minegaki et al. [55] aimed to investigate the anti-inflammatory effects of MRJP3 and its derived peptides both in vitro and in vivo since in vitro testing revealed that MRJP3 had anti-inflammatory activities. The addition of MRJP3 or its C-terminal tandem pentapeptide repeats (TPRs) sequence was shown to inhibit the expression of both TNF-α and IL-6 mRNAs in LPS-stimulated THP-1 cells. TPRs were injected into mice that suffered from herpes stromal keratitis (HSK) caused by the herpes simplex virus type 1 (HSV-1), the main objective consisting in the decrease of both disease scores and levels of TNF-α and IL-6 expression. Additionally, in both in vivo and in vitro models, the expression of TNF-α and IL-6 was decreased by TPRs derived from synthetic pentapeptides. 

The study conducted by Fatmawati et al. [56] evaluated endogenous antioxidant expression of nuclear-related factor 2 (Nrf2), transcription factor (Nf-kB), and pro-inflammatory cytokine TNF-α to determine the effectiveness of RJ as a UV radiation protector. Wistar rats were exposed for 2 h daily to 40 Watt UV-B lamps for a period of two weeks. RJ cream was applied in different concentrations, including 2.5%, 5%, and 10%, the highest dose inducing an increased Nrf2 and a decreased Nf-kB expression level. Moreover, TNF-α expression was significantly reduced with an increased RJ dose. As a result, the application of RJ cream shielded the skin from UV rays by reducing inflammatory reactions and strengthening cellular antioxidants.

Nanoparticles, such as nano-silver (NS), have the potential to cause inflammation by activating immune cells, which causes the release of pro-inflammatory cytokines. The skin, cardiovascular system, gastrointestinal and respiratory tract can all be negatively affected by NS. These adverse effects, including DNA oxidation and cell cytotoxicity, can be alleviated by using tissue-protecting compounds, such as RJ, along with NS. Thus, Pourmobini et al. [57] aimed to investigate RJ’s protective effect against NS’s inflammatory effect. Pro-inflammatory cytokines IL-1β, IL-2, IL-6, and IL-33 levels were measured in the kidney and liver of rats treated with NS, RJ, or a combination of NS and RJ. This study highlights the possibility that when combined, RJ and NS might have anti-inflammatory effects and affect immune cell activity. Therefore, combining RJ with NS may alter NS’s impact on the production of important pro-inflammatory cytokines, which indicates the need for more research on this topic in the future. 

The goal of the Salashoor et al. [58] study was to evaluate the anti-inflammatory and protective properties of RJ against ischemia/reperfusion (I/R)-induced renal diseases. Forty male rats were randomly assigned to one of four groups: placebo (0.9% saline), I/R, RJ (treated for 15 days with 300 mg/kg/day RJ), and I/R + RJ (pretreated for 15 days with 300 mg/kg/day RJ). Their study demonstrated that RJ has a protective effect against damage caused by I/R, perhaps due to its antioxidant and anti-inflammatory activities.

In the research conducted by Lin et al. [59], the focus lies in exploring the variations in the wound-healing properties of RJ produced by *Apis mellifera* L. during different blossom seasons of various floral sources. Their primary objective was to provide guidelines for the future rational application of RJ in cutaneous wounds based on the findings. Additionally, the study seeks to contribute to the further discovery of substances that promote wound repair. In this study, RJ samples collected during the flowering seasons of *Castanea mollissima* BL. (chestnut) and *Brassica napus* L. (rapeseed) in South China were investigated. Hydrophilic and lipophilic fractions were extracted from these samples. The wound-healing potential of the RJ samples was assessed in vivo using Wistar rats with excisional full-thickness wounds. The mechanisms of action were further explored through in vitro assays using human epidermal keratinocytes and macrophages stimulated with lipopolysaccharide (LPS). The results revealed distinct wound-healing properties among the different RJ samples. *Castanea mollissima* BL. RJ demonstrated higher potency, significantly accelerating wound closure between day 2 and day 4 with a rate of 0.25 cm^2^/day (*p* < 0.05). It also enhanced the proliferative and migratory capabilities of keratinocytes by 50.9% (*p* < 0.001) and 14.9% (*p* < 0.001), respectively. Furthermore, it modulated inflammation by inhibiting nitric oxide (NO) production by 46.2% (*p* < 0.001) and promoting cell growth through increased secretion of transforming growth factor-β (TGF-β1) by 44.7% (*p* < 0.001). On the other hand, *Brassica napus* L. RJ exhibited anti-inflammatory effects by reducing tumor necrosis factor-α levels by 21.3% (*p* < 0.001). Based on these findings, the study highlights the potential of *Castanea mollissima* BL. RJ for treating challenging wounds [59]. A randomized, double-blind trial conducted by Petelin et al. [60] aimed to find out how RJ supplementation affects oxidative and inflammatory indicators, as well as the metabolic profile of asymptomatic overweight people. Total cholesterol and inflammatory marker C-reactive protein both showed statistically significant declines after RJ supplementation, whereas anti-inflammatory marker adiponectin showed an increase. This investigation has pointed out that the daily use of 666 mg of lyophilized RJ has favorable effects on overweight people’s lipid profiles, inflammation, and antioxidant capacity. 

The purpose of the research conducted by Chansuwan et al. [61] was to explore the potential anti-inflammatory and anti-allergic properties of RJ-derived proteins and their enzymatic hydrolysates in mammalian cell lines. The bioactivity of protein hydrolysates depends on different factors, including peptide sequences and amino acids. Alcalase, Flavourzyme, and Protamex, three commercial proteases used to hydrolyze RJ, all possess anti-inflammatory and anti-allergic effects without altering the survival of macrophages and mast cells. Findings suggested that the protease type and hydrolysis duration may have an impact on their capacity to inhibit NO generation and -HEX release; Flavourzyme hydrolysates display the highest activity for both characteristics. Their anti-oxidative qualities and DNA damage-protecting action may be related to the anti-inflammatory impact. These findings indicate that RJ hydrolysates may be further developed into functional food and components for anti-inflammatory and anti-allergic activities.

The inhibition of RAW264.7 cell expression of the inflammatory gene was analyzed by Uthaibutra et al. [1]. RT-PCR technique was used to investigate inducible nitric oxide synthase (iNOS), COX-2, and IL-6 cells. Total RNA was isolated from the cells, and following a three-hour RJ treatment, they were stimulated with LPS. The outcomes demonstrated that LPS might activate inflammatory genes, and the expression of the iNOS gene could be suppressed at doses of 5 and 10 mg/mL RJ-LP1 and RJ-CM1.

## 5. Other Bioactive Effects of RJ

RJ is noteworthy for having a large number of bioactive substances, such as MRJPs and 10-HDA, which are considered to be the basis of all the valuable effects of bee-derived secretion [30].

**Figure 2:**
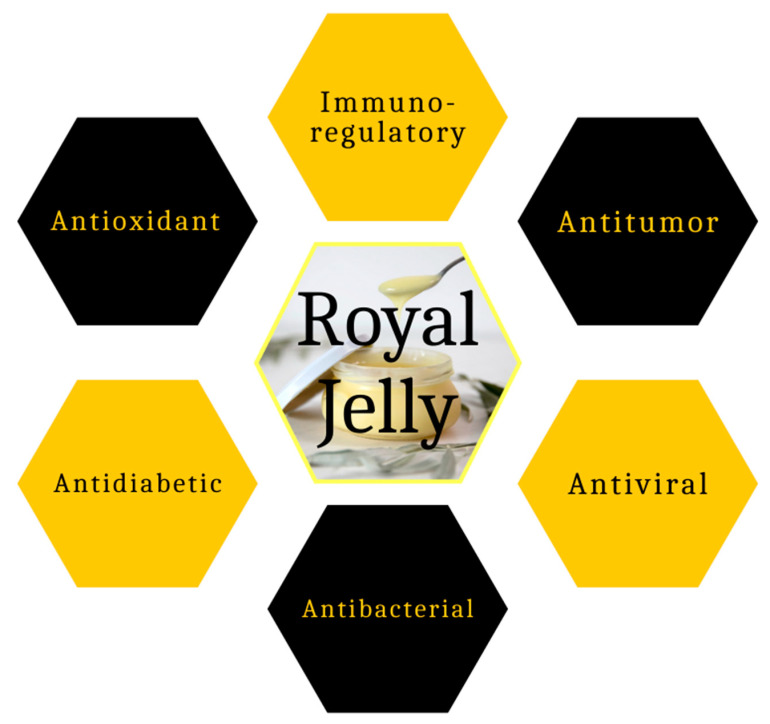
Other bioactive properties of RJ taken into discussion. (Created with canva.com, accessed on 9 April 2023).

Figure 2 presents other biological effects of RJ that this review takes into discussion, other than its anti-inflammatory one. These six properties of the food of the queens will be briefly discussed in the following paragraphs and table.

Some recent investigations suggest that RJ has potent antibacterial properties [6,57]. Antibiotic-resistant microorganisms are threatening the health of people across the world. Finding and creating modern antibacterial drugs to stop their spread is therefore becoming increasingly popular. Bee-derived products, such as RJ, honey, and propolis, are regarded as a natural alternative to synthetic antibiotics since they are rich in bioactive chemicals. RJ is renowned for combating deadly illnesses caused by a series of pathogenic agents in both humans and animals [27].

The antibacterial property of RJ has been tested on various bacterial strains, such as *Escherichia coli*, *Staphylococcus aureus*, *Pseudomonas aeruginosa*, *Listeria monocytogenes*, *Prevotella intermedia*, *Porphyromonas gingivalis*, *Aggregatibacter actinomycetemcomitans*, *Fusobacterium nucleatum* [27,62] and many more. Some authors pointed out that this antimicrobial effect appears due to RJ’s most important bioactive compounds: proteins, peptides, and 10-HDA [62]. Moreover, due to its minimal adverse effects, RJ can be viewed as a potential substitute for synthetic antibiotics [63]. Thus, RJ contains bioactive substances, including proteins, peptides, and fatty acids, which provide significant antibacterial effects against a variety of infections [27]. In other words, RJ can be regarded as a natural antibiotic with additional health benefits due to its unique chemical profile.

A wide range of studies have shed light on the antioxidant property of RJ, among its therapeutic benefits. Biomolecules essential for a healthy life can be degraded as a result of oxidative stress caused by the increased synthesis of ROS. This process is linked to the emergence of several pathogenic processes and chronic disorders. Researchers have emphasized the many benefits of employing antioxidants to treat and help prevent these conditions [30], and there has been an increase in RJ use in this sense in recent years. Thus, RJ may very well be able to play the part of a free-radical scavenger, according to a growing body of research [21]. In all of the investigations, including RJ compounds, their antioxidant property was expressed by favorable outcomes in a number of representative criteria [21,30]. Furthermore, according to available data, the radical-scavenging activity of RJ was proven by both in vitro and in vivo experiments, which confirmed the value of hive products in alleviating the negative consequences of oxidative stress and the illnesses caused by it (e.g., diabetes, atherosclerosis, neurodegenerative disorders, cancer) [21].

It has also been reported that bee products, such as RJ and bee bread, have anticancer properties [64]. Scientists and medical experts have worked together to develop a number of approaches that are still in use today in the battle against cancer, such as chemotherapy or radiotherapy. Nevertheless, the majority of these treatment strategies have severe drawbacks. It is well documented that several bee-derived compounds can aid in the suppression of cancer. More precisely, processes such as cancer cell proliferation, metastasis, and tumorigenesis inhibition have been discovered and linked to the antitumor effect of bee secretion [30]. Thus, there are studies that confirm RJ’s antitumor property and the fact that this bee product is a contender as far as complementary therapies for cancer go [65].

Moreover, RJ influences the production of various chemokines and growth factors, as well as the expression of cancer-related molecules in patients with malignancies, particularly in those treated with cytostatic drugs. It also reduces cell growth and activates cell death in malignant cells. RJ is hence believed to have anti-cancer effects on tumor development and to have protective qualities against toxic medication side effects [2]. For example, via the control of several cancer-related pathways, RJ and one of its key constituents, 10-HDA, can reduce tumor development and the migration of malignant cells [2].

In general, the research on the antitumor effects of RJ showcases this hive product as a potential source of cytotoxic chemicals with a variety of antitumor effects and mechanisms [64]. In addition, when RJ or its components are used alongside anticancer medication, their effects on the disease are synergistic, increasing the drug’s efficacy and even minimizing its negative effects. Recent investigations have provided strong evidence that RJ has an anticancer impact, whether used alone or in conjunction with other traditional cytostatic medications [66].

Furthermore, RJ presents various other health-boosting effects, including immunoregulatory, antiviral, and antidiabetic effects [30]. Studies that have explored the three aforementioned properties of RJ or some of its key components are briefly presented in Table 1.

## 6. Conclusions

Recent research articles have highlighted that RJ represents a valuable resource for treating inflammatory diseases, owing to its distinct pharmacological and therapeutic properties. The anti-inflammatory effects of RJ have been extensively investigated and substantiated by various experimental models and clinical studies. RJ has been shown to modulate key inflammatory mediators, including cytokines, chemokines, and adhesion molecules, thereby mitigating the inflammatory response. It exerts its anti-inflammatory actions through diverse mechanisms, such as inhibition of pro-inflammatory cytokine production, suppression of NF-κB signaling pathway, and modulation of immune cell function. These findings highlight the potential of RJ as a natural remedy for managing inflammation and related disorders. Although further research, including well-designed clinical trials, is warranted to establish optimal dosage, treatment regimens, and long-term safety, the existing scientific evidence underscores the potential of RJ as a valuable natural remedy for inflammatory diseases and warrants further exploration of its clinical applications.

## Figures and Tables

**Table 1:** Mechanisms underlying the immunoregulatory, antiviral, and antidiabetic effects of RJ.

Effect	Key Players	Mechanism	Type of Study	References
Immuno-regulatory	RJ	↓ total proteins and immunoglobulins, ↑ plaque-forming splenocytes, ↑ antibody production, and immunocompetent cell proliferation	In vivo (CBA mice)	[67]
	MRJP3	Suppression of IL-4 production, inhibition of serum anti-OVA IgE and IgG1 levels	In vivo (Mice)	[68]
	RJ	Innate immunity modulation through IIS/DAF-16, p38 MAPK, and Wnt signaling pathways	In vivo (*C. elegans*)	[69]
	RJ	↓ inflammation, ↑ cell regeneration	In vivo (Rats)	[70]
	MRJPs	Positive effects on immunoglobulin content, immune factor level, and proliferation of spleen lymphocytes	In vivo (Mice)	[71]
	RJ	Modulation of immune responses via downregulation of NLRP1	Clinical (human patients)	[72]
	RJ	Induction of antibody production, maturation of immune cells, stimulation of the innate and adaptive immune responses	Review	[73]
Antiviral	RJ	Inhibition of HSV-1	In vitro (Vero cells)	[74]
	MRJP2 and MRJP2 isoform X1	Sialic acid hydrolysis, attachment prevention (high binding affinity to viral receptor-binding sites), inhibition of SARS-CoV-2 enzymes, prevention of hemoglobin attack	In vitro (WI-38 lung cells)	[75]
	Erlose, Kaempferol glucoside, Iridin, Luteolin glucoside (Cynaroside)	Antiviral affinity (binding to COVID-19 binding sites through H-bonding), blocking of SARS-CoV-2 protease (through hydrogen bond and π-π T-shaped interactions)	In vivo and in vitro tests	[76]
Antidiabetic	RJ-propolis	Hypoglycemic and antioxidant activity	In vivo (Mice)	[77]
	RJ-honey	↓ plasma VLDL-C and TG, normalization of glycemic control indices	In vivo (Rats)	[78]
	RJ	↓ FBS, ↓ HbA1c, ↓ HOM A-I R, improvement in serum levels of triglycerides, cholesterol, HDL, LDL, VLDL, and Apo-A1, ↓ oxidative stress, ↑ antioxidant enzymes	Review	[78]
	RJ	Glycemic regulation (fasting blood glucose and glucose clearance as the most affected parameters)	Review	[78]

↓ decreasing effect; ↑ increasing effect.

## Data Availability

Not applicable.
